# The Influence of Interspecific Competition and Host Preference on the Phylogeography of Two African Ixodid Tick Species

**DOI:** 10.1371/journal.pone.0076930

**Published:** 2013-10-09

**Authors:** Nídia Cangi, Ivan G. Horak, Dmitry A. Apanaskevich, Sonja Matthee, Luís C. B. G. das Neves, Agustín Estrada-Peña, Conrad A. Matthee

**Affiliations:** 1 Evolutionary Genomics Group, Department of Botany and Zoology, Stellenbosch University, Matieland, South Africa; 2 Department of Veterinary Tropical Diseases, Faculty of Veterinary Science, University of Pretoria, Onderstepoort, South Africa; 3 United States National Tick Collection, The James H. Oliver, Jr. Institute for Coastal Plain Science, Georgia Southern University, Statesboro, Georgia, United States of America; 4 Department of Conservation Ecology and Entomology, Stellenbosch University, Matieland, South Africa; 5 Centro de Biotecnologia-UEM and Faculdade de Veterinária, Eduardo Mondlane University, Maputo, Mozambique; 6 Department of Parasitology, Veterinary Faculty, Zaragoza, Spain; University of Minnesota, United States of America

## Abstract

A comparative phylogeographic study on two economically important African tick species, *Amblyomma hebraeum* and *Hyalomma rufipes* was performed to test the influence of host specificity and host movement on dispersion. Pairwise AMOVA analyses of 277 mtDNA COI sequences supported significant population differentiation among the majority of sampling sites. The geographic mitochondrial structure was not supported by nuclear ITS-2 sequencing, probably attributed to a recent divergence. The three-host generalist, *A. hebraeum*, showed less mtDNA geographic structure, and a lower level of genetic diversity, while the more host-specific *H. rufipes* displayed higher levels of population differentiation and two distinct mtDNA assemblages (one predominantly confined to South Africa/Namibia and the other to Mozambique and East Africa). A zone of overlap is present in southern Mozambique. A mechanistic climate model suggests that climate alone cannot be responsible for the disruption in female gene flow. Our findings furthermore suggest that female gene dispersal of ticks is more dependent on the presence of juvenile hosts in the environment than on the ability of adult hosts to disperse across the landscape. Documented interspecific competition between the juvenile stages of *H. rufipes* and *H. truncatum* is implicated as a contributing factor towards disrupting gene flow between the two southern African *H. rufipes* genetic assemblages.

## Introduction

Successful dispersal and the subsequent ability to reproduce with conspecifics are central to maintaining the integrity of sexually reproducing species [1]. The factors affecting the dispersal of parasites, and how genetic material is mixed among geographic regions, are not well documented. Ectoparasites in particular provide a case in point [2] and contemporary phylogeographic uncertainties can partly be ascribed to the complex mode of ectoparasite reproduction, coupled to an incredible diversity in life forms [3]. For example, blood-feeding ixodid ticks (Acari: Ixodidae) comprise approximately 700 extant species [4,5] characterized by divergent life histories (two or three hosts needed to complete the life cycle; host specialists versus generalists; mating on or off the host etc.). In multi-host taxa, the different life stages also often parasitize a variety of vertebrate hosts (mammals, birds, reptiles and amphibians) with different abilities for dispersal [1].

Identifying the factors influencing the dispersal and genetic connectivity among tick populations of different geographic origin is thus not a trivial exercise and several hypotheses have been proposed. Key to the discussions are host specificity and the mobility of the hosts of adult ticks [1,6,7], the number and type of host species needed to complete the life cycle [8,9], sex-biased dispersal of different life stages [6,10], parasite-host immunity interactions [11] and abiotic factors associated with biogeographic barriers and other environmental changes [12–16].

A better understanding of the dispersal ability of ticks is essential since they constitute an economically important group of arthropods that act as vectors of diseases to domestic livestock and wild animals [10,17,18]. In an attempt to address the paucity of data needed to explain some of the mechanisms responsible for tick dispersion and gene flow, we selected two economically important African species, *Amblyomma hebraeum* and *Hyalomma rufipes*. *Amblyomma hebraeum* is responsible for the transmission of *Ehrlichia ruminantium* (the cause of heartwater in bovine species) [19,20], *Theileria mutans* (causing benign theileriosis in cattle) [21,22], and *Rickettsia africae* (causing African tick bite fever in humans) [23,24]. In the southern African context, *H. rufipes* is probably the most important vector of Crimean-Congo hemorrhagic fever (CCHF) virus to humans [25,26]. It also transmits *Anaplasma marginale*, the causative organism of bovine anaplasmosis [27], *Babesia occultans*, the cause of benign babesiosis in cattle [17,28] and *Ricketsia conorii*, the cause of tick typhus in humans [29].

**Figure 1:**
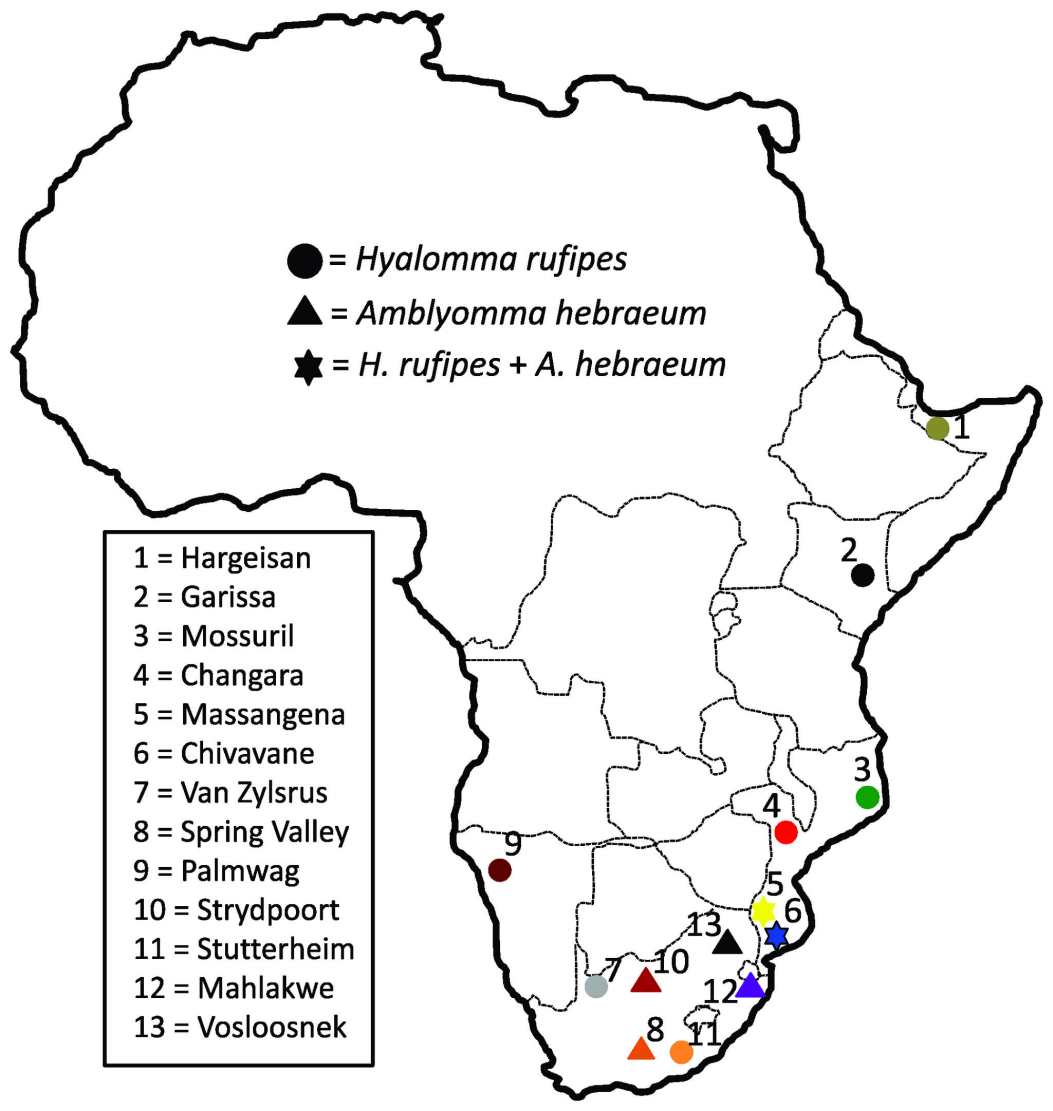
Sampling localities for *A. hebraeum* and *H. rufipes* used in the present study. Locality names correspond to those provided in Table 1 and colours correspond to those provided in Figures 2 and 3.

*Hyalomma rufipes* and *A. hebraeum* exhibit differences in life history traits, and when examined in a comparative fashion across a broadly speaking similar abiotic landscape, the data may help to explain some of the mechanisms involved in tick dispersion. The two species have partially overlapping distributions in southern Africa (Figure 1) and their adults mainly utilize highly mobile wild and domestic bovids as hosts [30–32]. It is thus predicted that the reproductively active adult stages of both species have the potential to frequently disperse over large tracts of land via natural host movement that could further also be facilitated by anthropogenic activities such as the trade in domestic animals. The life cycles of the two species differ, however, when the immature stages are taken into consideration. The larvae and nymphs of the three-host *A. hebraeum* feed on a wide variety of large and small mammals, including hares, ground-frequenting birds and sometimes tortoises [32–35] In contrast, the immature stages of the two-host tick, *H. rufipes* are host restricted and have only been recorded on hares and ground-frequenting birds [34,36–38]. In comparison, *H. rufipes* can thus be regarded as a more specialized parasite since the availability of wildlife is critical for the completion of its life cycle [37,39]. In *A. hebraeum* all three life stages can feed on the same adult host species [32,40] and this characteristic makes it a more generalist species with higher ecological plasticity [41].

Following from the above, it can be hypothesised that localized geographic populations of *H. rufipes* will exhibit stronger signals of population differentiation than *A. hebraeum*. To test this, we embarked on a comparative phylogeographic study to infer genetic connectivity among different sampling sites across the landscape. Ticks were sampled throughout southern and east Africa and were analyzed phylogeographically by making use of the mitochondrial COI gene, and in the case of *H. rufipes*, also the nuclear ITS-2 gene fragment. It was envisaged that the outcome of our investigation could provide new empirical data that can potentially be used to explain some of the mechanisms that play a role in the dispersal of ticks. This may in turn have direct implications for a better understanding of ectoparasite evolution and in particular speciation [42]. Should distinct genetic lineages be observed, the results of our study will also have medical/veterinary implications that will need further testing (for example the implementation of stricter control measures for the movement of domestic livestock especially if acaricide resistance can be coupled to genetic lineages) [18].

## Materials and Methods

### 2.1 Specimen collections

A total of 115 adult *A. hebraeum* were collected from six localities in southern Africa and 162 adult *H. rufipes* were sampled from nine localities (Figure 1; Table 1). Most specimens were collected from cattle, *Bos taurus*/*indicus* hosts and in the rare event that sufficient numbers of ticks were not available at a specific site, they were collected from sheep, *Ovis aries*, or goats, *Capra aegagrus*. No ethical clearance was required to perform work on invertebrate parasites and all ticks were collected with permission from landowners. Ticks were stored in 70% ethanol, and their specific identification was confirmed by Ivan Horak.

**Table 1:** Locality information and genetic summary statistics for the geographic samples included in this study.

Location	GPS Co-ordinates	N	H_n_ (H_s_)	h (± SD)	π (± SD)	Fu’s F_s_ (P value)	τ
*A. hebraeum*							
Massangena	21°32'0.0"S 32°57'0.0"E	20	6 (1)	0.64 (±0.12)	0.002 (0.001)	-2.394 (0.023)	0.994
Chivavane	25°3'0.00"S 33°37'60.00"E	20	7 (3)	0.79 (±0.06)	0.002 (0.001)	-2.908 (0.013)	1.375
Vosloosnek	25°0’0.0"S 30.5°0’0.0"E	18	5 (2)	0.67 (±0.08)	0.001 (0.001)	-1.822 (0.035)	1.016
Strydpoort	27°0’0.0"S 26° 0’0.0"E	18	7 (4)	0.82 (±0.06)	0.002 (0.001)	-2.521 (0.030)	1.516
Mahlakwe	27.5°0’0.0"S 32.5°0’0.0"E	20	4 (2)	0.28 (±0.13)	0.000 (0.000)	-2.749 (0.001)	3.000
Spring Valley	32°17'0.0"S 26°25'0.0"E	19	3 (2)	0.20 (±0.12)	0.000 (0.000)	-1.804 (0.012)	3.0
**All**		**115**	**22 (14)**	**0.66 (±0.08)**	0.002 (0.001)	**-23.366 (0.000)**	**1.056**
*H. rufipes*							
Hargeisan	9°33’44.60″N 44°04’37.25″E	19	15 (9)	0.96 (±0.04)	0.013 (±0.007)	-4.163 (0.040)	1.598
Garissa	0°27'28.83"S 39°39' 30.0"E	20	16 (11)	0.98 (±0.02)	0.009 (±0.005)	-7.286 (±0.003)	3.121
Mussuril	14°57'57.60"S 40° 39'39.97"E	18	9 (7)	0.80 (±0.09)	0.012 (±0.007)	1.035 (0.702)	18.682
Changara	16°25'25.7″S 33°37'4.3"E	20	16 (6)	0.97 (±0.03)	0.017 (±0.009)	-3.571 (±0.072)	1.346
Massangena	21°32'0.0"S 32°57'0.0"E	20	12 (2)	0.94 (±0.03)	0.009 (±0.005)	-1.879 (0.194)	1.625
Chivavane	25°3'0.00"S 33°37'60.00"E	19	12 (3)	0.92 (±0.05)	0.013 (±0.007)	-1.083 (0.310)	1.830
Stutterheim	27°0’0.0"S 26° 0’0.0"E	19	3 (0)	0.51 (±0.12)	0.001 (±0.001)	1.211 (0.753)	0.000
Van Zylsrus	26°20'18.28"S 22°36'44.03''E	8	5 (3)	0.86 (±0.11)	0.002 (±0.002)	-1.358 (0.100)	2.029
Palmwag	19°28'52.86"S 14°10'58.87''E	19	10 (6)	0.85 (±0.07)	0.006 (±0.003)	-2.106 (0.137)	2.879
**All**		**162**	**64 (47)**	**0.96 (±0.04)**	**0.009 (±0.005)**	**-24.096 (0.000)**	**0.486**

Locality designations correspond to those given in Figure 1. N is the number of mtDNA sequences; H_n_ is the number of haplotypes followed by (H_s_ = singletons); h is the haplotype diversity (± standard deviation); π is the nucleotide diversity (± standard deviation); Fu’s Fs and τ is also indicated (see text for details).

### 2.2 DNA extraction, PCR and sequencing

Genomic DNA was extracted from individual ticks following the manufacturer’s protocol for animal tissues using a DNeasy® Blood & Tissue kit (QIAGEN^TM^, Crawley, UK). Proteinase K (20mg/µl) digestion at 56°C was extended to 48 hours and the extracted DNA was eluted in 250µl of AE buffer and stored at -20°C.

A ~800 base pair stretch of the mitochondrial COI region was amplified by polymerase chain reaction (PCR), using a tick specific forward AR-U-COIa (5’-AAACTRTKTRCCTTCAAAG-3’) and reverse primer AR-L-COIa (5’-GTRTTAAARTTTCGATCSGTTA-3’), respectively (Ropiquet et al. unpubl. data). For the nuclear DNA, a portion of the ITS-2 was amplified by PCR from selected individuals of *H. rufipes*, using the tick specific forward primer RIB-8 (5’-GTCGTAGTCCGCCGTC-3’) and the reverse primer RIB-11 (5’-GAGTACGACGCCCTACC-3’) [43]. PCR reactions were performed following standard techniques with primer annealing at 40°C for COI and 62.5°C for ITS-2. PCR products were separated on 1% agarose gels stained with ethidium bromide and visualized using a UV light. After purification with the Nucleoafast 96 well plate-kit (Macherey-Nagel, Düren, Germany), PCR products were sequenced using the protocol prescribed by the BigDye terminator v 3.1 kit (Applied Biosystems, Warrington, UK)

### 2.3 Sequence data analyses

Sequences were manually inspected and edited with the software BioEdit v 7.0.5 [44]. The mitochondrial sequences were translated into proteins (http://www.ebi.ac.uk/Tools/emboss/transeq/index.htm) in order to ensure that there were no stop codons. Species authenticity was further confirmed with BlastN searches (http://blast.ncbi.nlm.nih.gov/Blast.cgi). The nuclear DNA haplotype reconstruction was conducted using DnaSP v 5.10 [45] and the default settings in PHASE v 2.1 [46] with 1 000 000 iterations.

Haplotype diversities (*h*), and nucleotide diversities (π) were estimated in Arlequin v 3.5 [47]. Uncorrected sequence divergences (distance matrix) were obtained using PAUP 4.0 [48]. Sequences were collapsed to haplotypes using DnaSP v 5.10 [45] and evolutionary relationships among haplotypes were depicted by statistical parsimony haplotype networks generated in TCS 1.21 [49]. Genetic differentiation between the various sampling populations was determined using an analyses of molecular variance (AMOVA) implemented in Arlequin v 3.5. By utilizing the outcome of the COI haplotype networks as priors, we also performed a hierarchical analysis of molecular variance (AMOVA [50]) to determine the level of variation within and among geographic groupings. Significance was estimated at the 0.05 level with 10 000 permutation steps.

As a complement to the traditional population genetic analysis (φ_ST_; F_st_), a model-based Bayesian clustering method was used to investigate the genetic structure across the geographic range (BAPS v 5.3 [51]). Analysis of “spatial clustering of individuals” and “groups of individuals” were performed without any pre-defined assumptions on geographic group structure. Each analysis was performed 10 times with different k values (K=1-10). To investigate geographic distance as a potential isolating mechanism, the Mantel test [52], as implemented in Arlequin v 3.5 [47] was used to test for isolation-by-distance.

In order to obtain information about processes that could have caused observed genetic variation, historical demography of populations were analyzed using two approaches. Firstly, to test for selective neutrality, Fu’s Fs [53], was estimated in Arlequin v. 3.5 [47]. Mismatch distributions were then used to test for population expansion [54–56] in Arlequin v. 3.5 [47]. Approximate time of population expansion, t, was calculated by substituting values for τ and μ in the equation τ = 2µt, where τ is given by the mismatch analysis as an estimate of the time of occurrence of the hypothetical expansion and μ (the mutation rate per site per generation) of 0.75% between ancestor-descendent alleles (i.e., half of 1.5%, the average value for arthropod pairwise differences per million years [57,58]) was used. τ is expressed in generations (months), while the value of μ is measured in years.

### 2.4 Climate suitability analyses

We assessed the suitability of climate for permanent populations of *H. rufipes* from collection records obtained between 1985 and 2010. The ‘Maximum Entropy Approach’ within the Maxent computer program for modelling species geographic distributions (v.3.3.3k [59]) was employed. Maxent is a general-purpose program that generates inferences from incomplete information, estimating a target probability distribution by finding the probability distribution of maximum entropy, subject to a set of constraints that represent the incomplete information concerning the reported distribution. Maxent is a machine learning modelling method, which has recently attracted attention because of its favorable performance in comparison to other modelling methods [60]. All models were produced with default parameter settings that are suited to a range of presence-only datasets [61]. The documented distribution of the species was obtained from [62] and records were updated with the most recent taxonomic overview of taxa [63]. We downloaded a set of monthly values of temperature and the Normalized Derived Vegetation Index (NDVI), at 0.1° spatial resolution (NEO-NASA web server, March 2000 to September 2011). NDVI was used as a proxy for humidity since this factor has been shown to be important for the survival of ticks. Reliability of the model was determined using the area under the ROC curve (AUC [64]). Models with values above 0.75 are considered potentially useful [65].

## Results

**Figure 2:**
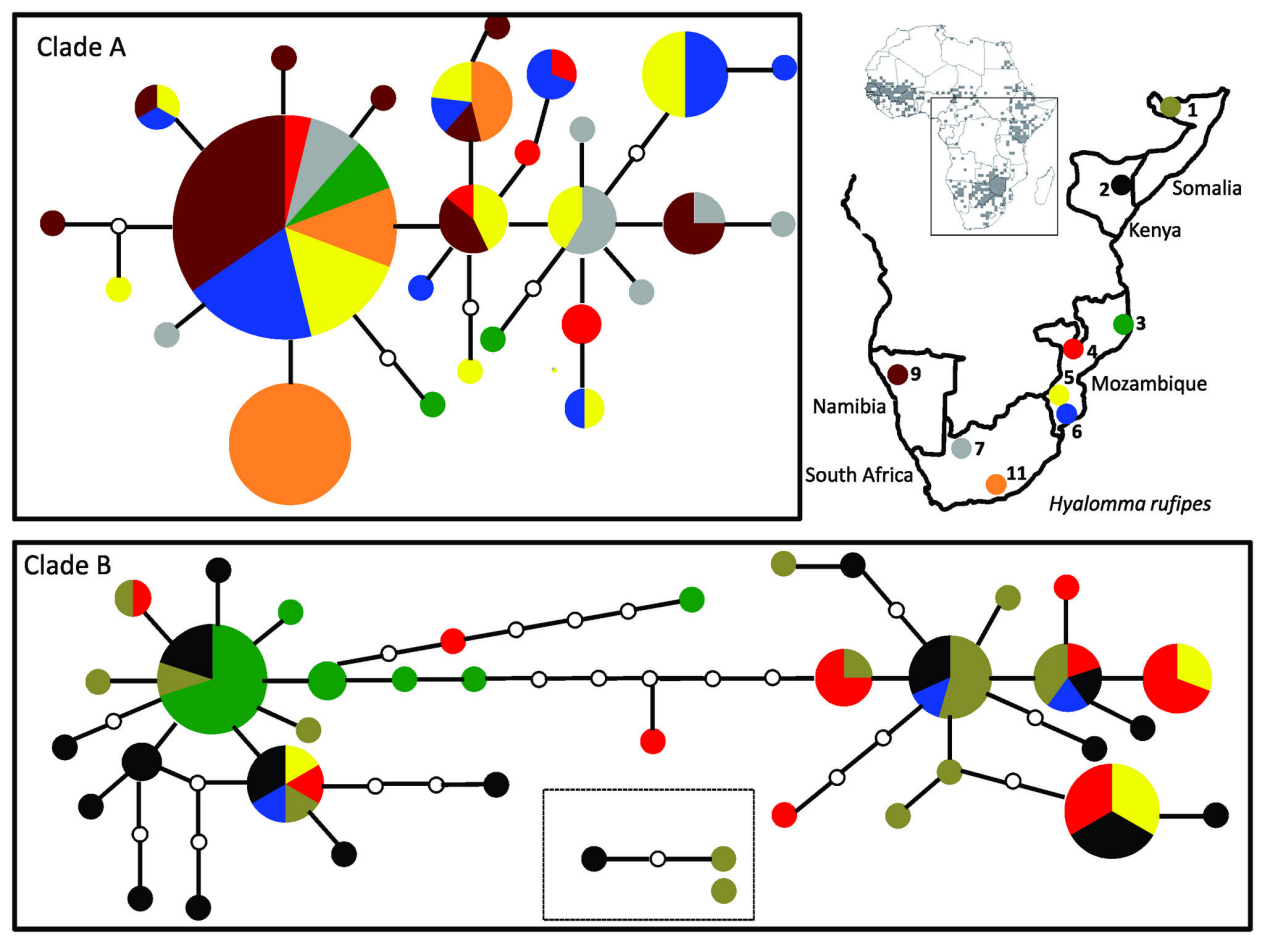
Haplotype network of the 22 mitochondrial COI haplotypes detected from *A. hebraeum.* The size of the circles corresponds to the number of individuals characterized by the specific haplotype. Each line separating haplotypes represents one mutational step and missing/intermediate haplotypes are shown by an white circle. Each sampled haplotype is color coded according to the sampling sites indicated on the map inset. Distribution map for *A. hebraeum* taken from [41,81].

**Figure 3:**
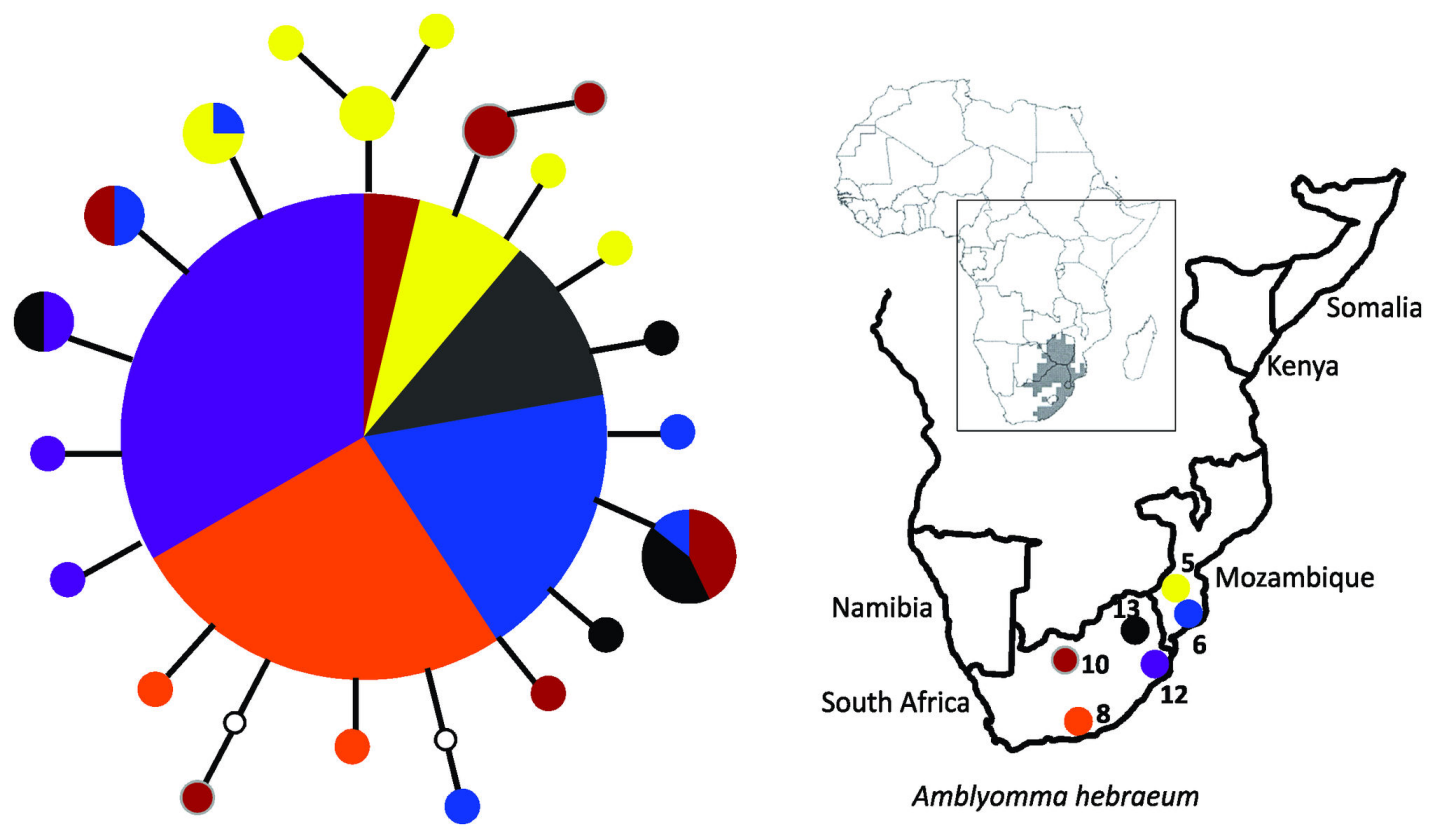
Haplotype network of the 64 mitochondrial COI haplotypes detected for *H. rufipes.* The southern and northern clades are indicated by solid boxes and the three unconnected haplotypes from north Africa is indicated in the form of a dotted box. The size of the circles corresponds to the number of individuals characterized by the specific haplotype. Each line separating haplotypes represents one mutational step and missing/intermediate haplotypes are shown by an white circle. Each sampled haplotype is color coded according to the sampling sites indicated on the map inset. Distribution map for *H. rufipes* taken from [41,81].

The 115 adult *A. hebraeum* individuals revealed 22 haplotypes for the mitochondrial COI gene fragment (Genbank Accession numbers: JX049245 - JX049266; Table 1; Figure 2). More than 50% of the haplotypes were unique to sampling localities resulting in a haplotype diversity (h) of 0.66 (Table 1). Marked differences in haplotypic diversity were detected among sampling sites and ranged from 0.20 in Spring Valley to 0.82 in a population from Strydpoort. The overall nucleotide diversity (π) was 0.002 (Table 1) and a similar low value was also reflected in the average sequence divergence value between the haplotypes (0.005%; ±0.0025). For *H. rufipes*, a total of 162 individuals were analyzed and 64 mtDNA COI haplotypes were identified (Genbank Accession numbers: JX049267 - JX049330; Table 1; Figure 3). The overall haplotype diversity (h) of 0.96 was much higher than that detected for *A. hebraeum* and ranged between 0.51 in a population from Stutterheim to 0.98 for the Garissa sampling site. Although low, the overall nucleotide diversity (π) of 0.009 was higher than that found for *A. hebraeum* and ranged between 0.001 at Stutterheim and 0.017 at Changara (Table 1).

A star-shaped statistical parsimony network for *A. hebraeum* was obtained reflecting a complete lack of geographic population structure, and a recent common ancestry for nearly all the maternal gene lineages (Figure 2). The most common haplotype was present in all sampled sites, representing 57% of the total number of individuals. In sharp contrast, the same analyses for *H. rufipes* resulted in two divergent statistically unconnected haplogroups (Clade A and B; Figure 3). Clade A will be referred to as Southern group and amongst others contains all the Namibian and South African individuals sampled at Palmwag, Van Zylsrus, and Stutterheim. Clade B will be referred to as the Northern group and contains haplotypes predominantly sampled at the Mozambique locations and further north in Africa. Interestingly, all the sample sites in Mozambique had some level of haplotype sharing between the two clades with the eastern Changara, Massangena and Chivane sampling sites showing larger amounts of admixture than the western Mussuril sampling site. No haplotypes belonging to Clade A were detected in Somalia and Kenya, and in fact, three haplotypes originating from Somalia and Kenya could not be connected to any of the other *H. rufipes* haplotypes detected in our study (indicated by the dotted line in Figure 3). The average uncorrected sequence divergence between the two clades is 3.17% (± 0.38%), with sequence diversity values of 0.70% (± 0.38%) within the Southern and 1.17% (±0.64%) within the Northern clade. Within the Southern clade there is no evidence for any geographic substructure, but within the Northern assemblage, two subgroups are present separated by at least 6 mutational steps from each other. The Bayesian analyses of population structure confirms the presence of a single lineage within *A. hebraeum* and two main assemblages within *H. rufipes* (K= 2). The analysis clusters one of the Mozambique sampling sites (Changara) within the Southern assemblage.

**Figure 4:**
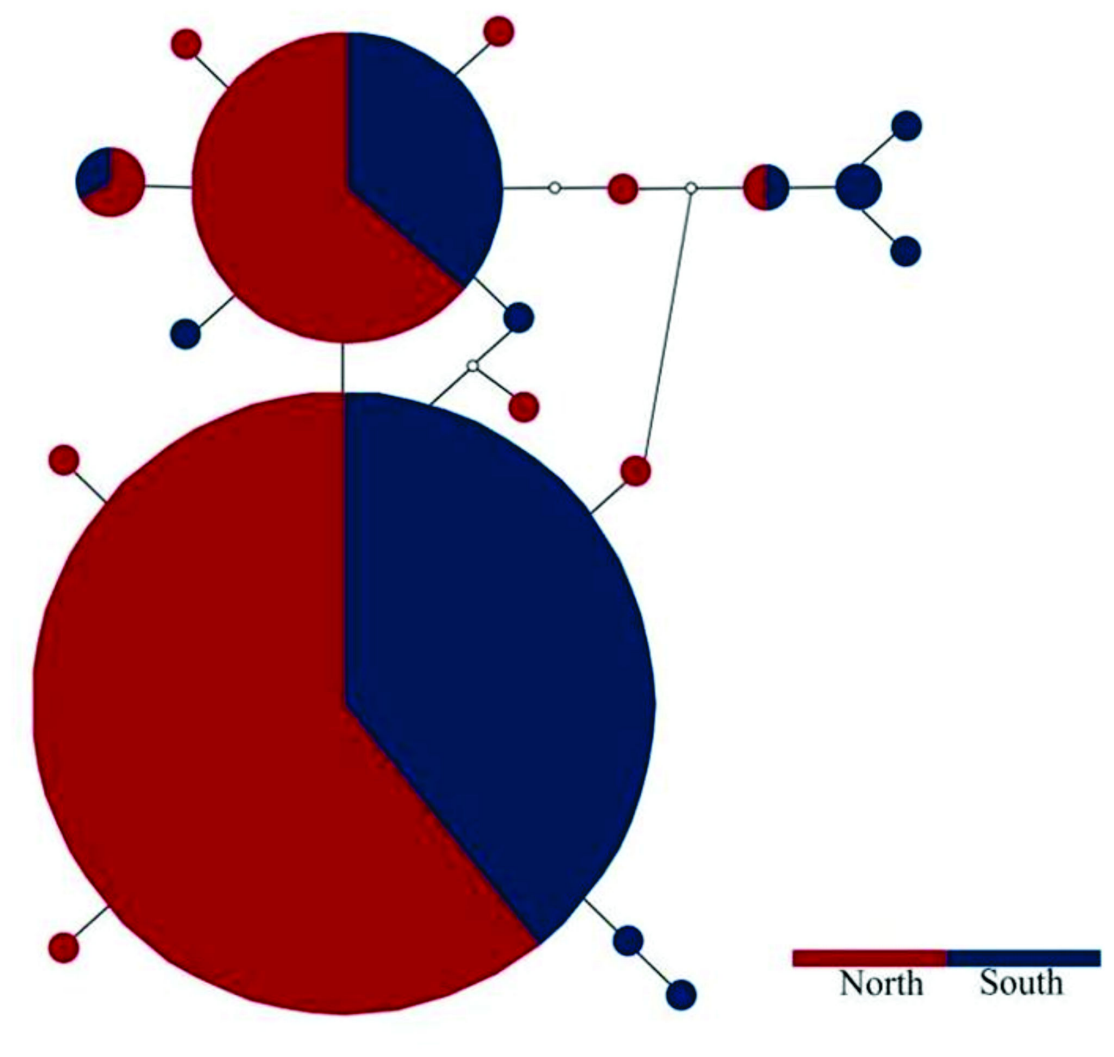
Haplotype network of the 19 ITS-2 haplotypes detected for *H. rufipes.* The size of the circles corresponds with the number of individuals represented by the haplotype. Each line separating haplotypes represents one mutational step and missing/intermediate haplotypes are shown by an white circle. For visual comparison between data sets, localities were color coded based on the outcome of the mtDNA analyses (southern clade = red; northern clade = blue) and these are also similarly indicated on the map inset.

Because of the low level of variation among *A. hebraeum* haplotypes, and the complete absence of mtDNA geographic structure for this species, only *H. rufipes* was targeted for nuclear ITS-2 sequencing. The 64 *H. rufipes* haplotypes retrieved by the mitochondrial COI analyses were amplified. Three individuals failed to amplify resulting in a total of 122 nuclear alleles available for the analyses. Nineteen nuclear haplotypes were retrieved in DnaSP v 5 (Genbank Accession numbers, JX049226 - JX049244) with an overall haplotype diversity (h) of 0.618 (±0.039) and an overall nucleotide diversity of 0.005 (±0.003). Contrary to the mitochondrial DNA analysis, the statistical parsimony network resulted in one haplogroup for all the alleles with no evidence of geographic structure (Figure 4).

AMOVA analysis for *A. hebraeum*, detected shallow but significant genetic structure, with an overall Φst value of 0.196 (p < 0.05). Contrary to the expectations based on the star-shaped haplotype network, pairwise Φst comparisons between geographic localities were significant in most instances supporting some degree of population differentiation across the range (Table 2). A similar suite of geographic population differentiation analyses for the more specialized *H. rufipes* also revealed significant genetic structure among most sampling sites (Table 3), and a higher degree of differentiation overall (Φst value of 0.43; p < 0.05). It is possible that the higher level of differentiation can simply be due to differences in sampling extent. When the analyses were performed partitioning the individuals in Southern and Northern assemblages (as defined by the BAPS analysis), 66% of the variation can still be ascribed to among populations within clades (p < 0.05). AMOVA analysis of the ITS-2 data was performed by specifying the populations according to the mtDNA groups and this resulted in a low (0.001) and non significant Φst value between groups.

**Table 2:** Population pairwise Φst matrix of *A. hebraeum* among sampling regions generated by AMOVA.

	Massangena	Chivavane	Vosloosnek	Strydpoort	Mahlakwe	Spring Valley
Massangena	0.0					
Chivavane	0.164	0.0				
Vosloosnek	0.055	0.230	0.0			
Strydpoort	0.199	0.317	0.074	0.0		
Mahlakwe	0.032	0.216	0.148	0.325	0.0	
Spring Valley	0.032	0.222	0.165	0.333	0.0	0.0

Significance values are in bold (p < 0.05). Locality names correspond to Table 1.

**Table 3:** Population pairwise Φst matrix of *H. rufipes* among sampling regions generated by AMOVA.

	Hargeisan	Garissa	Mussuril	Changara	Massangena	Chivavane	Stutterheim	Van Zylsrus	Palmwag
Hargeisan	0.0								
Garissa	0.0	0.0							
Mussuril	0.076	0.067	0.0						
Changara	0.121	0.195	0.104	0.0					
Massangena	0.532	0.613	0.493	0.228	0.0				
Chivavane	0.408	0.492	0.365	0.106	0.0	0.0			
Stutterheim	0.735	0.807	0.714	0.489	0.231	0.291	0.0		
Van Zylsrus	0.642	0.737	0.615	0.350	0.021	0.099	0.455	0.0	
Palmwag	0.638	0.716	0.607	0.353	0.011	0.094	0.217	0.0	0.0

Significance values are in bold (p < 0.05). Locality names correspond to Table 1.

The Mantel test (Mantel 1967) for the mtDNA data showed no relationship between genetic and geographic distances for *A. hebraeum* (r = 0.0, p = 0.6) and a similar scenario existed when each clade of *H. rufipes* was analyzed separately (Northern group: r = 0.25, p = 0.2; Southern group: r = 0.27, p = 0.1). For both *A. hebraeum* and *H. rufipes* Fu’s Fs was negative and significant (Table 1) and subsequent mismatch analysis for *A. hebraeum* revealed a unimodal distribution of pairwise differences that is consistent with a recent population expansion model (SSD = 0.002, p = 0.2; Table 1). Likewise, when the two *H. rufipes* assemblages were analyzed individually, each one produced a unimodal distribution of pairwise differences, and these were not significantly different from a population expansion model (Southern clade: SSD= 0.032, p= 0.4; Northern clade: SSD= 0.001, p= 0.6). To compare the potential effect of the demographic history on diversity estimates of the two tick species, the entire mtDNA data sets for the two species were used respectively to calculate the time since expansion for each. Given τ = 1.056 for *A. hebraeum* and τ = 0.486 for *H. rufipes* the time since expansion is 70 400 years ago for *A. hebraeum* (approximately 1 year of generation time [66]) and 64 800 years ago for *H. rufipes* (6 months of generation time [67]).

**Figure 5:**
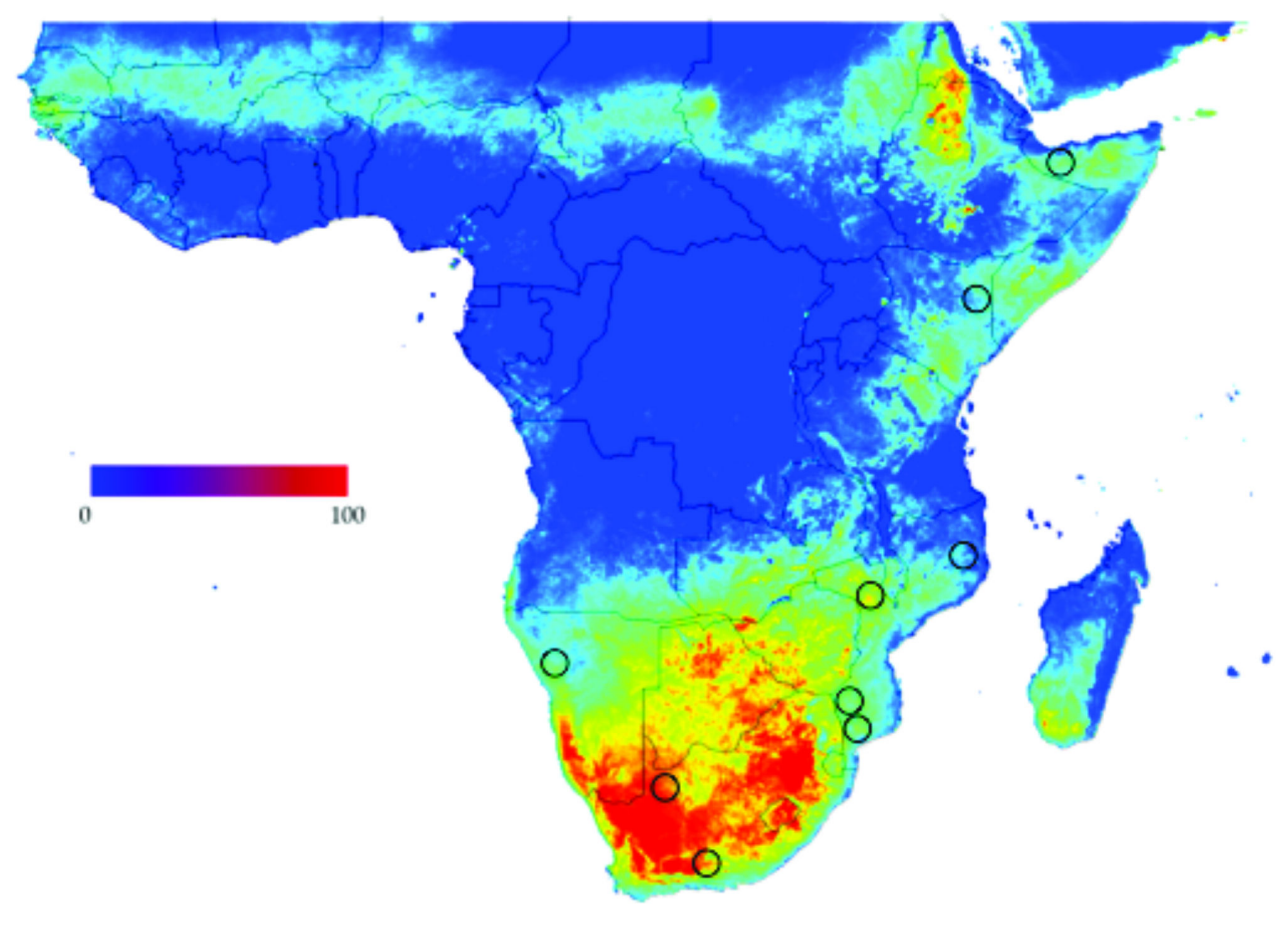
Climate suitability of *H. rufipes* in Africa based on temperature and the Normalized Derived Vegetation Index (NDVI), at 0.1° spatial resolution. Suitability are indicated in a sliding window where red are most suitable and blue are least suitable. Sampling localities are indicated by black circles.

*H. rufipes* shows large areas of high climate suitability in Africa (Figure 5). The overall AUC for the best model is approximately 0.8. Areas of rain forests in central Africa and the Sahelian and sub-Saharian zones are not regarded as suitable climates for the tick. Pertinent to the focus of our study, however, the two *H. rufipes* clades are not separated by a strong zone of “non suitable” habitat, suggesting that some alternative factor plays a role in the disruption of mtDNA gene flow in the southern part of the species range.

## Discussion

The broad-scale phylogeographic patterns obtained for *A. hebraeum* and *H. rufipes* is markedly contrasting. The absence of any geographic structure in the host generalist, *A. hebraeum*, suggests a single geographic/taxonomic unit while the strong mtDNA genetic partitioning in the host specialist, *H. rufipes*, provides new evidence for allopatric evolutionary trajectories. The latter is supported by the complete mtDNA reciprocal monophyly of the two *H. rufipes* clades (Figure 2), coupled to a relatively narrow zone of overlap in the south east of Mozambique (predominantly at localities 4, 5 and 6; Figure 1). The lack of noticeable morphological differences among individuals belonging to the two mtDNA clades (Horak pers observation), and the lack of resolution at the more conservative nuclear ITS-2 level (Figure 4), could reflect an incipient speciation processes (with the retention of ancestral polymorphisms [68]). Our study, however, also suggests that a third *H. rufipes* lineage may exist in North Africa (3 individuals from Kenya and Somalia could not be linked to the geographically proximate Northern clade; Figure 3). More intensive sampling from West and North Africa will be needed to reach any firm taxonomic conclusions, especially in the light of the report that *H. rufipes* can hybridize with *H. truncatum* and *H. dromedarii* in this region [43].

The comparative phylogeographic patterns obtained in this study are useful towards gaining greater insights into the factors influencing the dispersal of ticks. A striking difference between the two species involves haplotypic and nucleotide diversity estimates (*A. hebraeum*: h = 0.66; π= 0.002; *H. rufipes*: h = 0.96, π= 0.009; Table 1). The differences in genetic diversity between the two lineages are also well illustrated when only the region of geographic overlap between the two species is considered (see structured complex haplotype network for clade A of *H. rufipes* versus the starlike haplotype network of *A. hebraeum*, *cf*. Figures 2 and 3). Numerous factors can be advanced as contributing towards this discrepancy. It is for example possible that differences in the evolutionary rate between the two species exist and or different selection pressures operate on the mtDNA lineages [69–71]. It can, however, also be argued that these results are simply due to the larger geographic range occupied by *H. rufipes*, but in the absence of significant isolation by distance (also see 72), this seems not to be the case. A more recent founder event for *A. hebraeum* can also result in low diversity values (also see 73), but the estimated times of population expansion for both species are roughly similar (70 400 years ago for *A. hebraeum* and 64 800 years ago for *H. rufipes*). We argue that the more pronounced genetic diversity and geographic structure obtained for *H. rufipes* is rather a result of the restrictions in their abilities to disperse (two host specialist needing wildlife to complete its life cycle) while the less structured pattern obtained in *A. hebraeum* supports a higher level of dispersal among geographic sampling sites (three-host generalist which can complete its entire life cycle on the same host).

Despite the marked differences in genetic diversity estimates, individual populations of both species exhibit a fairly high degree of differentiation among sampling sites (Tables 2 and 3; also see 72,74–77). This finding is counterintuitive given the wide variety of highly mobile hosts that can facilitate the dispersal of adult ticks of both species [32–38,78]. In addition, domestic cattle are frequently moved over large distances for anthropogenic reasons [31,76,79]. Furthermore, the immature stages of both species are often reported on birds [34,37], of which several species that may be infested with *H. rufipes* are migratory [36,38]. Host movement is thus not the main contributing factor driving the differentiation among sampling sites. It seems more plausible to suggest that the perceived pattern is rather due to capacity of the immature stages of the ticks to survive off the host in the various microhabitats [80–82]. Since the engorged female ticks detach from their hosts, and lay eggs in a sheltered environment, the availability of suitable hosts for the immature stages seems crucial towards ensuring the completion of the life cycle. Indeed, the structure obtained in the more specialized two-host tick, *H. rufipes* (which is dependent on wildlife to complete its life cycle [83],; and is more sensitive to desiccation in the environment [84],) is more pronounced than that found in the more habitat tolerant generalist *A. hebraeum* (where all three life stages can utilize the same domestic or wild host to complete its life cycle [32]).

If the physical environment, and particularly the availability of suitable hosts for the immature stages, play an important role in the phylogeographic structure of these two tick species, it is interesting to speculate on the reason/s for the two distinct mtDNA genetic clades found in *H. rufipes* (Figure 2). The observation that the co-distributed species, *A. hebraeum*, does not show a similar genetic break would support the idea that the two *H. rufipes* assemblages are probably not the result of a strong abiotic isolating event in the region (vicariance; also see 85). The projected range of spatial distribution of *H. rufipes* (based on temperature and the Normalized Derived Vegetation Index) suggest that the two genetic clades cannot be explained by unsuitable climate. Local host associations for different hare species, however, has been documented as an important factor for the successful completion of the life cycle of certain tick species [10] and even if suitable hosts are available, larvae well-adapted to harsh conditions can die from desiccation and starvation if a host is not found in time [86]. Since *A. hebraeum* seems to be more robust in withstanding harsh environmental conditions [84], and juveniles can survive on adult hosts also, the key to the difference in phylogeographic structure is probably in the availability of suitable hosts for the immature stages of *H. rufipes*.

At first site, the availability of a suitable host to the juvenile stages of *H. rufipes* does not seem to provide a plausible for obtaining two genetic assemblages in southern Africa. Hares acting as hosts for juvenile *H. rufipes* are abundant within the region where the genetic break has been observed (Kruger Park Region in South Africa and southern part of Mozambique). We thus propose that the structure could have been facilitated by a secondary enigmatic complexity related to competition between different tick species [37,87,88]. In the case of *A. hebraeum* its geographic distribution seems to be limited because of interspecific competition with *A. variegatum* [41,82,89]. For *H. rufipes*, the immature stages have to compete with H. truncatum for the predilection attachment site around the neck of hares [90]. This competition appears to be more intense in the western regions of South Africa [35,90,91]. On the other hand, in the south-eastern region of South Africa, *H. rufipes* is virtually exclusively present on hares as hosts for their immature stages [90]. However, in the Kruger National Park (bordering Mozambique) there is a complete absence of immature stages of *H. rufipes* larvae on scrub hares and in this this region, they are replaced by *H. truncatum* [35,90,92]. Given that the Kruger National Park is situated parapatrically to the zone of contact between the two *H. rufipes* assemblages, it is thus plausible to speculate that competitive habitat exclusion on hosts for immature stages may severely limit gene exchange across this region, and thereby play a further role in the genetic differentiation between the two *H. rufipes* assemblages.
